# A “data-driven uncertainty” computational method to model and predict instabilities of a frictional system

**DOI:** 10.1186/s40323-023-00241-3

**Published:** 2023-02-13

**Authors:** Farouk Maaboudallah, Noureddine Atalla

**Affiliations:** Department of Mechanical Engineering, Groupe d’Acoustique de l’Université de Sherbrooke (GAUS), Sherbrooke University, Sherbrooke, Quebec, J1K 2R1 Canada

## Abstract

Most of the recently developed methods for predicting instabilities of frictional systems couple stochastic algorithms with the finite element method (FEM). They use random variables to model the uncertainty of input parameters through standard probability laws. Regardless of the fact that advanced numerical schemes are available nowadays, a systematic and accurate method to describe finely the uncertainties upstream the model, and thus predict its response is still missing. In this contribution, we present a data-driven stochastic finite element scheme to predict the dynamic behavior of a rubbing system. The proposed framework relies on data-driven approach and uses four steps. In the first, the measured data are integrated directly, for the uncertainty quantification, by means of the random balance design (RBD). In the second step, the generated stochastic data are evaluated in an iterative way to solve friction-induced vibration problem. In the third step, the resulted data are reordered in such a way that the corresponding values of each measured input parameters are ranked in ascending order. Finally, the Fourier spectrum is introduced on the reordered results to compute the sensitivity indices. Thus, instead of Monte Carlo-based formalism or Fourier Amplitude Sensitivity Test (FAST), the computational cost of the proposed method is kept down to $\mathcal {O}(N)$ with *N* the number of samples. We investigate the efficiency of the suggested solver on a reduced brake system. Altogether, the suggested procedure achieves excellent accuracy at a much reduced computational time compared to the methods available in the literature.

## Introduction

The study and prediction of instabilities in frictional systems remain, to this day, an important and open issues in both academic and industrial research. Over the last decades, there have been many formulated theories [1–8], experimental investigations [9–15] and advanced models [16–21] with the aim of understanding the physics of the squeal phenomenon. In spite of this, friction-induced vibrations remains one the unresolved engineering problems where the consistency between predictions and experiments is often questioned.

Traditionally, the modelling and prediction of the squeal phenomena are carried out using deterministic solvers by means of the finite element method (FEM). Among the intense debates concerning the appropriate deterministic method for predicting friction-induced vibrations, there is the one that evokes the comparison between (i) the direct transient analysis (DTA) and (ii) the complex eigenvalues analysis (CEA) [20, 22–24]. It turns out that the two solvers, in a deterministic context, do not reproduce the squeal frequency observed experimentally [12]. The DTA method is more precise compared to the CEA. Indeed, the latter may lead to an underestimation or an over-estimation of the unstable modes observed in the nonlinear time simulation due to the fact that linear conditions (i.e. the linearized stability around an initial equilibrium point) are not valid during transient or self-excited oscillations [25]. On the other hand, CEA offers a good compromise between computational cost and accuracy. As a result, it is the most widely used in the industrial sector. To overcome the CPU cost of transient methods, authors have developed alternative deterministic approaches. For instance, Coudeyras et al. [26, 27] have proposed the Generalized Constrained Harmonic Balance Method (GCHBM) to compute nonlinear or pseudo-periodic responses of the frictional system. Charroyer et al. [28, 29] have combined the CEA with the shouting method to solve friction-induced vibrations. It should be noted that the aforementioned methods reproduce results of the DTA in a very reasonable computation time [26–29]. In parallel to this relevant studies, authors have show, in the most recent contributions, that the deterministic approach is not sufficient to predict instabilities because a frictional system (e.g. braking system) is subject to several sources of uncertainty [30–33]. Indeed, CEA or DTA, in a deterministic mode, represents only one case of reality. They neglect various uncertainties linked to manufacturing errors, rubbing contact, test conditions, etc. Naturally, these scientific challenges gave rise to the stochastic finite element method (SFEM), which attempts to couple the stochastic algorithm with FEM. The main challenge of these methods is to (i) model and (ii) propagate uncertainties upstream of the model, then to (iii) predict, using a probabilistic approach, dynamic instabilities. Among the various existing methods for quantifying uncertainty is the traditional Monte Carlo (MC) method, introduced by Culla et al. [19], to predic the instabilities. It was shown that MC-FE can improve the correlation between the prediction and the experiment [19, 34, 35]. In our previous work [36], we investigated the convergence of MC-FE solver by adding the classic variance-based sensitivity analysis (VBSA): it was shown that this method has a slow convergence rate. Hence, it is not efficient for this type of problem. In the same paper, the coupling between Fourier amplitude Sensitivity Test (FAST) algorithm with FEM demonstrated good convergence properties [36]. The polynomial chaos (PC) theory, originally developed by Ghanem et al. [37], is another popular class of methods to represent uncertainty with ambiguous information. Sarrouy et al. [38] and Nechak et al. [39] introduced both PC expansion and multi-element generalised polynomial chaos (MEgPC) to solve the stability of friction-induced vibrations. In the same context of the meta-modelling of instabilities, Denimal et al. [40] and Nechak et al. [41] applied the kriging method and coupled it with VBSA in Sobol’s sense. In the aforementioned advanced methods, authors mainly use the friction coefficient, the penalty coefficient of the contact interfaces and all material properties as random variables. Investigations have shown that the frictional contact parameters (i.e. friction coefficient and penalty coefficient) as well as the pad stiffness are the most sensitive predictors of instabilities [36, 42]. These results thus demonstrate the complex and mutliphysical side of friction-induced vibrations. Consequently, the uncertainty quantification of the previous dominant parameters should be taken into account for a robust prediction of the instabilities.

In this light, the direct stochastic simulations or meta-modelling techniques of friction-induced vibrations present a promising alternative to deterministic approaches that are merely available to handle single type of uncertain case through nominal input parameters. Usually, random variables are assumed to follow a standard probability density function (PDF). For instance, Zhang et al. [34] used Cauchy and Beta distributions, constructed using the parametric estimation, to model the friction coefficient. The consistency of such a method may be questioned if the size of the experimental sample is not sufficient to guarantee the convergence of the estimators. Another serious drawback of the stochastic scheme is the convergence. Indeed, a non-negligible number of stochastic iterations is required to estimate statistical indicators (e.g. partial variance, moments, etc.). In our recent study [36], two stochastic solvers are developed: (i) the traditional MC-FE and (ii) FAST-FE. The computational cost for the former solver is $N/2 \times (k+2)$ where *N* and *k* denotes the number of stochastic iteration and the number of random variables, respectively. With regard to the FAST-FE solver, a minimum of *N* iterations are needed to avoid the aliasing effect and interferences between random variables. Note that the stochastic iterations *N* depends indirectly on the number of random variables such that $N = 2 \times M \times \max (\omega _i) + 1$ where *M* is the harmonic order and $\omega _i$ is a characteristic frequency assigned to the *i*-th random variable. If the uncertainty analysis request several input parameters, the periodic sampling frequencies $\omega _i$ will be large. As a result, the model can become computationally prohibitive. For these reasons, stochastic modelling techniques for instabilities require further adaptation and enhancements to (i) reproduce and integrate data with unusual statistics (e.g. biased, multi-modal, etc.) and to (ii) optimize the CPU time for FAST-FE scheme.

To the best of our knowledge, the data-driven approach associated with stochastic finite element scheme to predict instabilities has so far received few attention. Thus, one of the objectives of the present contribution is to conduct a FAST-FE based numerical analysis of a rubbing system, capable of directly integrating the experimental measurement of the input parameters while ensuring a good convergence rate. The main scientific contributions of this document are the following:

- $\star$ Integrating the random balance design (RBD) within FAST-FE solver. Hence, the inherent criterion for avoiding aliasing effect between random variables is no more needed;
- $\star$ Constructing a data-driven random generator based on the RBD approach. The friction-induced problem is solved directly on a given set of measurement by bypassing the parametric estimation and the traditional PDF fitting;
- $\star$ Performing an efficient prediction of instabilities within a reasonable computational cost. The latter is completely dissociated from the number of random variables.

The rest of the paper is organized as follows. In “Data-driven stochastic complex eigenvalue analysis” section, the stochastic framework of the CEA method is formulated involving 3 modules (i) the stochastic pre-processing (ii) the model evaluation and (iii) the stochastic post-processing. In “Numerical treatment” section, the solver’s algorithm, illustrating the implementation of the data-driven approach to integrate the uncertainties upstream of the model and to predict its response, is presented opening the path to interface with an off-the-shelf computational engine (open source or not). The implementation strategy is then followed by numerical examples in “Numerical analysis of the suggested solver” section, where the solution of the proposed data-driven framework is compared to the solution of the methods available in the literature with regards to accuracy and efficiency. In the end, the paper closes with an application of the methodology to an industrial disc brake system followed by a conclusion and some perspectives.

## Data-driven stochastic complex eigenvalue analysis

In this section, we will introduce the stochastic CEA-based framework, developed in this paper, to predict the dynamic behavior of frictional system. The theoretical formulation of the proposed strategy will start with the stochastic pre-processing. The latter is devoted to enhance the periodic random generator of FAST algorithm by introducing the RBD approach with the possibility of data generation from experimental measurements. After model evaluation by the traditional CEA solver, the stochastic post-processing module will be presented to derive an estimate of the variance, the partial variance and the sensitivity index.

### Stochastic pre-processing

Let us consider $x = (x_1, x_2, \dots , x_n)$ a vector of *n* random variables each distributed according to a specific PDF $f_i(x_i)$, $i \in \{1, 2, \dots , n \}$. The input parameter space, called also the *x*-space, can be defined as:

1 $$\begin{aligned} \mathbb {K}^n_x = \{x = (x_1, x_2, \dots , x_n) \mid x_i \sim f_i(x_i), ~ i = 1, 2, \dots , n \}. \end{aligned}$$

In the version of FAST-FE solver developed in [36, 42], the original periodic sampling approach to simulate the random variable vector x [43–45]. The idea behind it is to carry out a bijective transformation (see Eq. (5)) between the *x*-space and the $\theta$-space defined by:

2 $$\begin{aligned} \mathbb {K}^n_{\theta } = \{\theta = (\theta _1, \theta _2, \dots , \theta _n) \mid -\pi< \theta _i < \pi , ~ i = 1, 2, \dots , n \}, \end{aligned}$$

where $\theta _i$ is a random variable distributed uniformly between $-\pi$ and $\pi$. Therefore, the generated data are sampled periodically in the $\theta$-space following a particular research curve function $G_i$, associated to the *i*-th random variable, which is the solution of the following equation,

3 $$\begin{aligned} {\left\{ \begin{array}{ll} \pi \sqrt{1-x^2} f_i(G_i(x)) \frac{d G_i(x)}{d x} = 1 ~ ,\forall i \in \{1, \dots , n\} \\ G_i(0) = \frac{1}{2} \end{array}\right. }. \end{aligned}$$

Without any loss of generality, let us assume a uniform random variable where its PDF is equal to 1, the corresponding research curve function, $G_i$, is deducted directly from Eq. (3). We write:

4 $$\begin{aligned} G_i(x) = \frac{1}{2} + \frac{1}{\pi } \arcsin (x). \end{aligned}$$

Note that the transition between the *x*-space and $\theta$-space is done periodically as follows,

5 $$\begin{aligned} x_i = G_i(\sin (\theta _i)). \end{aligned}$$

In the general framework where the random variable $x_i$ is modelled by its cumulative distribution function (CDF), $F_i$, the research curve function becomes:

6 $$\begin{aligned} x_i = G_i(\sin (\theta _i)) = F^{-1}_i \left( \frac{1}{2} + \frac{1}{\pi } \arcsin (\sin (\theta _i)) \right) . \end{aligned}$$

Note that in Eq. (6), $F^{-1}_i$ denotes the inverse cumulative distribution function (ICDF) of the *i*-th random variable.

Sampling in $\theta$-space, according to the traditional FAST algorithm, makes use of another bijective transformation, $\zeta$, defined in Eq. (8). Indeed, for each uniform random variable $\theta _i$, a unique characteristic frequency $\omega _i$ and grid sample $s_i$ are assigned in order to generate data in $\theta$-space. In other words, the random variable $\theta _i$ is sampled in another space, called the grid space $\mathbb {K}^n_s$ defined by:

7 $$\begin{aligned} \mathbb {K}^n_{s} = \{s = (s_1, s_2, \dots , s_n) \mid s^{(j)}_i = \frac{\pi }{N} + \frac{2\pi }{N}(j-1) - \pi , ~ j = (1,2, \dots , N) \}, \end{aligned}$$

over *N* drawn samples.

8 $$\begin{aligned} \theta _i = \zeta (\omega _i s_i) \end{aligned}$$

Now, since all the transformations are considered, the discretized form of the periodic sampling approach for a given random variable $x_i$ characterized by its CDF is given by:

9 $$\begin{aligned} x^{(j)}_i = F_i^{-1}\left( \frac{1}{2} + \frac{1}{\pi } \arcsin (\sin (\omega _i s^{(j)}_i))\right) , \end{aligned}$$

where *j* denotes the sample element.

It should be noted that for the traditional FAST algorithm, the characteristic frequency set $\{\omega _i \}$, $i = 1, \dots , n$ is selected according to a complex algorithm to avoid any kind of interference or aliasing effect, between the random variables $x_i$, up to a given order *M* of harmonics. In this case, the search curve is said to be space-filling. As a result, a criterion that must be respected is formulated. This restriction takes the form of a lower limit on the number of samples $N_{\text{ min }}$, for FAST-FE solver, which must be strictly observed. We write,

10 $$\begin{aligned} N_{\text{ min }} = 2 M \max {\{\omega _i\}} + 1. \end{aligned}$$

However, if the dimension of the input parameter space increases, the quantity $\max {\{\omega _i\}}$ increases as well. Therefore, the CPU times will be prohibitive.

In this context, we introduce the RBD approach within FAST-FE solver. Following this approach, all the random variables will be sampled with the same characteristic frequency $\omega$. Without any loss of generality, let us assume that $\omega = 1$ for all random variables. Equation (9) can be rewritten as,

11 $$\begin{aligned} x^{(j)}_i = F_i^{-1}\left( \frac{1}{2} + \frac{1}{\pi } \arcsin (\sin (s^{\beta _i^{(j)}}_i))\right) , \end{aligned}$$

with $\beta _i$ denotes a random permutation of the set $\{1,2,\cdots ,N\}$. Hence, $s^{\beta _i}_i$ reads as a random permutation of the auxiliary variable $s_i$ assigned to the random variable $x_i$. Hereafter, if not mentioned differently, the auxiliary variable vector $s = (s_1, \dots , s_i, \dots , s_n)$ is constructed from a “new” grid space,

12 $$\begin{aligned} \mathbb {K}^n_{s} = \{s = (s_1, s_2, \dots , s_n) \mid s^{(j)}_i = s_0 + \frac{2\pi }{N}(j-1) -\pi , ~ j = (1,2, \dots , N) \}, \end{aligned}$$

where $s_0$ is a uniform random variable drawn from $[0, \frac{\pi }{N}]$. We write $s_0 = \mathcal {U}(0,\frac{\pi }{N})$.

Following the logic of the grid space $\mathbb {K}^n_{s}$ in Eq. (12), it can be seen that *N* points can be drawn over a subspace of $\mathbb {K}^n_{s}$ defined only by $\frac{2\pi }{N}(j-1) -\pi$ (i.e. the second part of the *j*-sample $s^{(j)}_i$) which is quite equivalent to the first definition of the grid space in Eq. (7). According to this, one can observe that the second part of *j*-sample in Eq. (12) and also the *j*-sample in Eq. (7) will always return the same point in $\mathbb {K}^n_{s}$. To overcome this drawback and thus, make the random generator more efficient, an independent uniform variable $s_0$ is added in the new formulation of $\mathbb {K}^n_{s}$. Thereupon, the *N* starting points will be everywhere within $\mathbb {K}^n_{s}$, exactly like the traditional sampling of the so-called Latin Hypercube Sampling.

Since the same frequency is used in the RBD approach (see Eq. (11)), a serious issue may arise. The latter concerns the “space-filling” condition of the research curve. In other words, the constructed random generator in Eq. (11) will cover only a subspace of the whole *x*-space $\mathbb {K}^n_x$, which can induce a bias in the estimates of stochastic indicators (especially the partial variances). To avoid this kind of problem, Tarantola et al’s implementation will be used to trace the signature of each random variable after being evaluated by the deterministic FEM [46]. Therefore, instead of using the periodicity of the model output, combined with the different frequencies $\omega _i$, to track the influence of the variable $x_i$ at $\omega _i$, random permutations $s^{\beta _i^{(j)}}_i$ of the grid space $\mathbb {K}^n_{s}$ will be performed to generate a set of scramble points that cover the whole input space $\mathbb {K}^n_{x}$. Thus, the influence of the parameter $x_i$ on the model output will be completed by an equivalent permutation to that of grid space. More details are discussed in “Stochastic post-processing” section.

Following the proposed pre-processing module, a question naturally arises: “How can the uncertainties observed experimentally be integrated efficiently into modelling techniques?” One intuitive approach often used in literature is the parametric estimation wherein the probability law is assumed to have a particular shape (e.g. Gaussian distribution). In this case, it is sufficient to estimate a few parameters (mean and variance) to describe it completely. On the other hand, information about the probability law is very limited without saying that it is almost non-existent. In very specific conditions, the available data that characterize random variables involves a non usual behavior (e.g. multi-modal behavior). Therefore, the parametric estimation using the usual PDF (or CDF) will fail to capture the physics. With Eq. (11) in hand and following the algorithm 1, the random data can be generated and thus integrated, in modeling processes, from raw sets that do not necessarily follow a known analytical distribution.

### Model evaluation: atability analysis—deterministic FEM

The output of the pre-processing module presented in “Stochastic pre-processing” section is a design of experiments (DOE). It represents *N* random generated data directly from the measurement raw sets or from the usual probability laws. For each drawn *j*-realization $\{ x^{(j)}_1, x^{(j)}_2, \dots , x^{(j)}_n \}$ with $j \in \{1, 2, \dots , N \}$, the problem becomes deterministic. Then, the traditional stability analysis (CEA) is performed. The goal is to predict unstable modes by solving the following equation of motions:

13 $$\begin{aligned} M^{(j)} \ddot{u}^{(j)}(t) + C^{(j)} \dot{u}^{(j)}(t) + K^{(j)} u^{(j)}(t) + F^{(j)}_{\text{ nl }}(u^{(j)}(t)) = F_{\text{ ext }}(t), \end{aligned}$$

where $u^{(j)}(t)$ denotes the displacement vector associated with the stochastic iteration *j*, the dot refers to its derivative with respect to time. $M^{(j)}$, $C^{(j)}$ and $K^{(j)}$ are the mass, damping and stiffness matrices constructed for the stochastic iteration *j*, respectively. $F^{(j)}_{\text{ nl }}$ describes the non linear normal and tangential contact forces. Finally, $F_{\text{ ext }}$ is the external load.

Under the framework of FEM, the resolution of Eq. (13) is done through 2 levels. First and under the quasi-static assumption, the following equation,

14 $$\begin{aligned} K^{(j)} u^{(j)}_e + F^{(j)}_{\text{ nl }}(u^{(j)}_e) = F_{\text{ ext }}, \end{aligned}$$

is solved in order to find the equilibrium position, noted by $u^{(j)}_e$, leading to an homogeneous linear system in Eq. (15), where $K_{\text{ nl }}$ is the so called Jacobian matrix of the non linear contact forces at the equilibrium state $u^{(j)}_e$. Notice that the global stiffness matrix, $K_{\text{ asy }}$, in Eq. (15) is asymmetrized by the contribution of the frictional contact. In this case, it is recommended to use the non-symmetric solver to ensure the convergence.

15 $$\begin{aligned} M^{(j)} \ddot{u}^{(j)}(t) + C^{(j)} \dot{u}^{(j)}(t) + \underbrace{ \left( K^{(j)}+K^{(j)}_{\text{ nl }}(u^{(j)}_e) \right) }_{K^{(j)}_{\text{ asy }}} u^{(j)}(t) = 0 \end{aligned}$$

Second, the deterministic CEA solves Eq. (16), obtained after modal reduction, to provide a local stability analysis which is quantified by the Laplace parameter $p^{(j)}_k$ and the complex eigenvector $\phi ^{(j)}_k$ for each mode *k*. Furthermore, the dynamic behavior of the frictional system becomes unstable around the equilibrium state $u^{(j)}_e$, if and only if at least one Laplace parameter has a strictly positive real part $\alpha ^{(j)}_k$. In this case, the friction-induced vibration instability occurs and is quantified by two quantities: (i) its eigenpulse $\omega ^{(j)}_k$ which corresponds to the imaginary part of Laplace parameter $p^{(j)}_k$ and (ii) its magnitude of the instabilities $\alpha ^{(j)}_k$.

16 $$\begin{aligned} \left( p^{(j)^2} M^{(j)} + p^{(j)} C^{(j)} + K^{(j)}_{\text{ asy }} \right) \phi ^{(j)} = 0 \end{aligned}$$

It should be noted that, for the instability quantification in braking system, authors in [9, 47] use another criterion, known as the tendency of instability (TOI), which may be expressed under the *j* stochastic iteration as follows:

17 $$\begin{aligned} \text{ TOI}^{(j)} = \displaystyle \sum _{k} \frac{\alpha ^{(j)}_k}{\omega ^{(j)}_k} \times 1000, ~~ \forall ~ \alpha _k > 0. \end{aligned}$$

The TOI index is strongly correlated with the negative damping of the frictional system. Therefore, an unstable system will have a significantly higher index, implying a negative damping below zero. This explains the dynamic instability and the self-excited character, leading to a divergence of the equilibrium state.

### Stochastic post-processing

After the model evaluation, a $N \times m$ size set of Laplace parameter *p* is computed, where *N* and *m* denotes the number of stochastic iterations and the number of the complex modes, respectively.

Without any loss of generality, let us consider Laplace parameter $p_k$ for a specific mode *k*. Then, we write the model output as follows,

18 $$\begin{aligned} p_k = \text{ FEM } (x^{(j)}_1, x^{(j)}_2, \dots , x^{(j)}_i, \dots , x^{(j)}_n), ~~ \forall j \in \{1, 2, \dots , N \}. \end{aligned}$$

Eq. (18) can be read as an evaluation of the generated DOE, $\{ x^{(j)}_1, x^{(j)}_2, \dots , x^{(j)}_n \}$, through the deterministic CEA.

Due to the RBD approach (see “Stochastic pre-processing” section), the model output $p_k$ must be reordered in such a way that the realizations of the random variable $x_i$ are ranked in an increasing order. In other words, Laplace parameter for the mode *k* should be rewritten respecting the same order as the auxiliary variable $s_i$ (assigned to the random variable $x_i$) defined in the grid space $\mathbb {K}^n_{s}$ in Eq. (12) before the so-called permutation $\beta _i$. According to the inverse permutation $\beta _i^{-1}$, the generated data as well as the model output $p_k$ can be reordered by solving the following problem:

$$\begin{aligned} \begin{aligned}&\underset{x_i}{\text {find}}{} & {} \beta _i^{-1} \\&\text {such as}{} & {} x_k^{\beta _i^{-1^{(j)}}} = F_k^{-1}\left( \frac{1}{2} + \frac{1}{\pi } \arcsin (\sin (s^{\beta _k \circ \beta _i^{{-1}^{(j)}}}_k))\right) \; i,k = 1, \ldots , n \\&\text {with}{} & {} \beta _k \circ \beta _i^{{-1}^{(j)}} = {\left\{ \begin{array}{ll} j &{} \text { if } k=i,\\ \beta _k \circ \beta _i^{-1^{(j)}} &{} \text { if } k \ne i, \end{array}\right. } \; ~~~~~~~~~~~ j = 1,\dots , N. \end{aligned} \end{aligned}$$

It is important to note that $\beta _k \circ \beta _i^{-1^{(j)}}$ is a non-trivial permutation involving the random variable vector *x* except $x_i$. In the following, the reordered Laplace parameter will be noted by $p^{\beta ^{-1}}_k$, with $\beta ^{-1}$ refers to the inverse permutation of the permutation vector $\beta = (\beta _1, \dots , \beta _n)$.

Since the periodic research curve is introduced to generate random data, both Laplace parameter $p_k$ and the reordered Laplace parameter $p^{\beta ^{-1}}_k$ for each mode *k* are forced to be a multiple periodic function over the vector $\theta = (\theta _1, \dots , \theta _n)$. Therefore, the FE model can be expanded into Fourier series as follows,

19 $$\begin{aligned} \Re (p_k^{\beta ^{-1}}) = \displaystyle \sum _{\gamma _1, \dots , \gamma _n = - \infty }^{+\infty } C^{(\theta )}_{\gamma _1, \dots , \gamma _n} e^{\textbf{j}(\gamma _1 \theta _1 + \dots + \gamma _n \theta _n)}, \end{aligned}$$

where the operator $\Re (.)$ refers to the real part of Laplace parameter $p_k$, $\textbf{j}$ denotes the imaginary unit and $C^{(\theta )}_{\gamma _1, \dots , \gamma _n}$ is the complex Fourier coefficient. We have,

20 $$\begin{aligned} C^{(\theta )}_{\gamma _1, \dots , \gamma _n} = \left( \frac{1}{2 \pi } \right) ^n \int _{-\pi }^{\pi } \ldots \int _{-\pi }^{\pi } \text{ FEM } \circ G (\theta _1, \dots , \theta _n) e^{-\textbf{j}(\gamma _1 \theta _1 + \dots + \gamma _n \theta _n)} \, d \theta _1 \dots d \theta _n. \end{aligned}$$

Note, in Eq. (20), the function *G* denotes the search curve defined in Eq. (4). It is also important to note that the same research curve is used to sample all the random variable $x_i$.

From Eq. (20), Fourier spectrum can be derived. Namely

21 $$\begin{aligned} \varLambda _k(\gamma _1, \dots , \gamma _n) = \Bigl |C^{(\theta )}_{\gamma _1, \dots , \gamma _n} \Bigr |^2. \end{aligned}$$

It should be pointed out that the Fourier spectrum $\varLambda _k$ is evaluated at $(\gamma _1, \dots , \gamma _n)$. The latter may contain the fundamental characteristic frequency $\omega$ and its harmonics up to order M. In this contribution, the fundamental frequency $\omega$ is kept at 1. However, the choice of the harmonic order *M* will be investigated in “Numerical analysis of the suggested solver” section.

Throughout Eqs. (20) and (21), some interesting stochastic quantities can be computed; (i) the variance of the model *V* and (ii) the partial variances $V_{x_i}$ for each random variable $x_i$. Recall that the latter aims to identify the effect of the input parameters on the predicted instabilities. Subsequently, we write,

22 $$\begin{aligned} V(\Re (p_k^{\beta ^{-1}})) = \displaystyle \sum _{ \mid \gamma _1 \mid , \dots , \mid \gamma _n \mid = 1}^{+\infty } \varLambda _k(\gamma _1, \dots , \gamma _n), \end{aligned}$$

and

23 $$\begin{aligned} V_{x_i} (\Re (p_k^{\beta ^{-1}})) = V(E[\Re (p_k^{\beta ^{-1}}) \mid x_i]) = \displaystyle \sum _{ \mid \gamma _i \mid = 1}^{+ \infty } \varLambda _k(0, \dots ,0, \gamma _i,0, \dots , 0). \end{aligned}$$

Thus, the sensitivity index for the random variable $x_i$ can be deduced directly using Eqs. (22) and (23), we write:

24 $$\begin{aligned} D_i(\Re (p_k^{\beta ^{-1}})) = \frac{V_{x_i} (\Re (p_k^{\beta ^{-1}}))}{V(\Re (p_k^{\beta ^{-1}}))}. \end{aligned}$$

Based on Eq. (24), the random variable $x_i$ with a high sensitivity index leads to a greater variance in the expected value of $\Re (p^{\beta ^{-1}}_k)$ given $x_i$. Thus, it indicates that a relatively large proportion of the so-called dynamic instability variance is contributed mainly by the random variable $x_i$. Knowing this information, one can (i) reduce or even eliminate the instabilities of the system and (ii) optimize the design chain of the frictional system by reducing the number of input parameters. In the following, we present a numerical treatment of the suggested data-driven approach to compute the quantities in Eqs. (22), (23) and (24).

## Numerical treatment

In sum, the following set of equations has to be solved for Laplace parameter *p*:

25 $$\begin{aligned} {\left\{ \begin{array}{ll} x = F^{-1}\left( \frac{1}{2} + \frac{1}{\pi } \arcsin (\sin (s^{\beta }))\right) ~~~ \forall x \in \mathbb {K}^n_x, ~ \forall s \in \mathbb {K}^n_s \\ \left( p^2 M(x) + p C(x) + K_{\text{ asy }} (x) \right) \phi = 0 ~~~~~~~~~ \forall p \in \mathbb {C}^m \\ D(\Re (p)) = \frac{V_{x} (\Re (p))}{V(\Re (p))} \\ \end{array}\right. } \end{aligned}$$

with *x* is a random variable vector. It may contain the distribution of Young’s modulus, the friction coefficients or the mass density following a given CDF vector *F*. The mass *M*, damping *C* and stiffness *K* matrices depend on the random variable vector *x*. The above matrices are of dimension *m* by *m*. Finally, a design mapping *D*, such as the introduced in [36], which involves the sensitivity indices defined above is used to synthesize the most probable unstable mode and visualize the effect of the random variable vector on the dynamic instability for each mode *k* in $\{1, \dots , m\}$.

### Pre-processing procedure

The preprocessing procedure proposed in this paper can simulate any given probability distribution. If the latter is usual (e.g. Gaussian), Eq. (11) can be used to generate the random variates. In the opposite case where the probability law is unknown, one can use directly a raw data set to derive the random generator. This implies that the suggested approach should adapt to the acquired information. To do so, a suitable algorithm 1 is proposed and contains 3 main steps:

1. *CDF estimation* $F_i$: Let $\{x_i^{1}, x_i^{2}, \dots , x_i^{N_e} \}$ represent $N_e$ observations of the random variable $x_i$. The correspondent empirical distribution function (EDF) estimation, $\hat{F}_i$, can be computed as follows,
  26 $$\begin{aligned} \hat{F}_i(t) = \frac{1}{N_e} \displaystyle \sum _{j=1}^{N_e} 1_{x_{i}^{j} < t} ~ \xrightarrow [N_e \longrightarrow + \infty ] ~ F_i. \end{aligned}$$
  Note that as long as $N_e$ tends towards $+\infty$, the EDF converges to the CDF almost surely whatever the value of *t* (strong law of large numbers statement).
2. *ICDF estimation* $F^{-1}_i$: Eq. (26) provides an non-derivable piecewise constant function. Instead of working on the latter, Newton’s scheme will be applied directly on a selection of the computed EDF points by ensuring their uniqueness. It should be noted that the convergence of the algorithm 1 is quadratic as long as the initial guess $X_0$ is near the solution.
3. *Construction of the random generator*: Knowing the ICDF derived from raw data, the random generator will be constructed using Eq. (11). Again, the grid space should be sampled through the definition in Eq. (12) followed by a random permutation $\beta$ to cover the whole input parameter space $\mathbb {K}_x^n$. In the following, the permutation of the vector grid sample *s* is denoted by $s^{\beta }$.

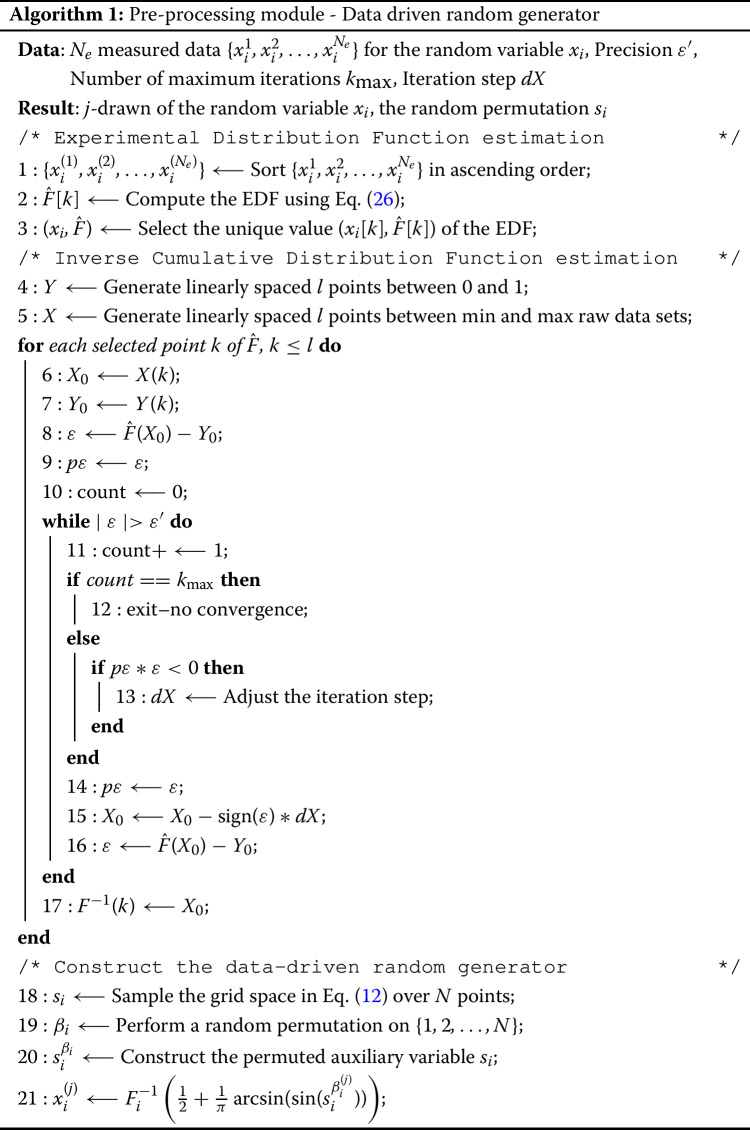


### Post-processing procedure

*N* realizations are generated through algorithm 1 accompanied with their random permutation $\beta$. Note that the latter denotes a vector of random permutations associated to the random variable vector. For each realization *j* of the random variable vector, the problem becomes deterministic. This leads to a traditional resolution by CEA through FEM in order to find the Laplace parameter for each mode. Upon solving the deterministic problem in Eq. (13) on the whole statistical population, the associated random complex eigenvalues are collected. From this point, one can compute the so-called stochastic moments using the classic estimators. In this paper, the most-likely unstable modes are computed using a probabilistic approach. Once done, the desired sensitivity information is deducted from the Fourier spectrum in Eq. (21). The computation of Fourier’s spectrum is obtained by switching to the grid space $\mathbb {K}^n_{s}$ through Eq. (8), thus the FE model can be expanded again over the auxiliary variable.

27 $$\begin{aligned} \Re (p_k) = \text{ FEM } \circ G (\zeta (\omega _1 s), \dots , \zeta (\omega _n s))^{\beta ^{-1}} = \displaystyle \sum _{l = - \infty }^{+\infty } C^{(s^{\beta ^{-1}})}_{l} e^{\textbf{j}(ls)}, \end{aligned}$$

with,

28 $$\begin{aligned} C^{(s^{\beta ^{-1}})}_{l} = \frac{1}{2 \pi } \int _{-\pi }^{\pi } \Re (p_k) e^{\textbf{j}(ls)} \, d s. \end{aligned}$$

It should be noticed that, in the RBD framework, the frequencies $\omega _i$ are kept to 1 as mentioned above. It is also important to notice, that the post-processing over the auxiliary variable should be done on the reordered model output. Thus, it is convenient to recover the inverse permutation $\beta ^{-1}$.

By using the rectangle rule with a rectangle width of $\frac{2 \pi }{N}$. Equation (28) can be discretized as follows,

29 $$\begin{aligned} \begin{aligned} C^{(s^{\beta ^{-1}})}_{l} = \frac{1}{2\pi } \int _{-\pi }^{\pi } \Re (p_k) \textbf{e}^{-\textbf{j} l (s)} \, d s&\\ \simeq \frac{1}{N} \displaystyle \sum _{j=1}^{N} \Re (p^{(j)}_k) \textbf{e}^{-\textbf{j} l (s^{(j)})}. \end{aligned} \end{aligned}$$

Following that, the variance of the model can be computed as,

30 $$\begin{aligned} \hat{V}(\Re (p_k)) = \displaystyle \sum _{\mid l \mid =1}^{N/2} \mid C^{(s^{\beta ^{-1}})}_{l} \mid ^2, \end{aligned}$$

and the partial variances can be estimated as,

31 $$\begin{aligned} \hat{V}_{x_i}(\Re (p_k)) = \displaystyle \sum _{\mid l \mid =1}^{M} \mid C^{(s^{\beta _i^{-1}})}_{l} \mid ^2. \end{aligned}$$

Recall that *M* is the harmonics order. A detailed study is devoted to this subject in “Effect of the harmonic order *M*” section to comment on the relevant choice of its value.

As long as the periodic function $\text{ FEM } \circ G$ is twice continuously differentiable, the error of the rectangle rule decays as the square of rectangle width. According to Davis and Rabinowitz [48], the numerical error of Fourier coefficient, $C_l^{s^{\beta ^{-1}}}$, reads

32 $$\begin{aligned} \varepsilon _{C_l} \le \frac{\pi ^3}{3 N^2} \max _{s \in [-\pi , \pi ]} \left( \frac{ \partial ^2 \text{ FEM } \circ G}{\partial s^{2}} \right) . \end{aligned}$$

Another interesting point is that the characteristic frequencies have been reduced to 1. This choice as well as the use of random permutations, allowed to ease the restrictions on the number of stochastic iterations. However, due to the so-called inverse permutation,

$$\beta _k \circ \beta _i^{-1^{(j)}}, ~ \forall k,i=\{1,\dots ,n\},$$

Fourier coefficient $C^{(s^{\beta _i^{-1}})}_{l}$ related to the random variable $x_i$ may contain two contributions; (i) the partial variance $V_{x_i}$ related to $x_i$ and (ii) a portion of the partial variance $V_{\sim x_i}$ for all random variables except $x_i$ (see AAppendix for more details). In other words, Eq. (31) may incorporate interference between the last partial variances, which may lead to an overestimation of the true value of the partial variances contributed by the main effect. The quantification of the last overestimation is provided in Appendix where the quality of Fourier spectrum estimation is evaluated. It has been found that the latter is biased. In fact, the residual contributed by the inverse permutation of all random variables except $x_i$ induces a bias of order $\frac{1}{\sqrt{N}}$. Hence, besides the error of the rectangle rules, the bias has to be handled. Furthermore, an accurate estimation of Fourier spectrum and thus the sensitivity index requires a high number of the stochastic iteration *N*. On the other hand, a high number of samples is subjected to an intensive CPU time. A compromise has therefore to be found between the accuracy and the computational cost.

Using the result of Appendix , see Eq. (59), we arrive at the estimation of the partial variance:

33 $$\begin{aligned} \hat{V}_{x_i} \approx V_{x_i} + \frac{\hat{V}_{\sim x_i}}{N}, \end{aligned}$$

where $V_{x_i}$ denotes the theoretical partial variance related to the main effect. $\hat{V}_{\sim x_i}$ represents the residual partial variance from the all variable expect $x_i$. It should be noted that if the number of stochastic iterations is small, the bias becomes important and hence, the computed partial variances or sensitivity indices will be under or over estimated. In the spirit of minimizing the error made by the bias (as shown in Eq. (33)) a correction is introduced, inspired from the work of [49], on the estimated partial variance $\hat{V}_{x_i}$. The former reads:

34 $$\begin{aligned} \hat{V}^c_{x_i} = \frac{\hat{V}_{x_i} - \frac{2 M}{N} \hat{V}}{1 - \frac{2M}{N}}, \end{aligned}$$

where $\hat{V}_{x_i}$ is the classic partial variance, $\hat{V}$ is the estimated variance, *M* is the harmonics order and *N* is the number of the stochastic iterations. Note that the introduced correction is activated only if the number of stochastic iterations is low. If not, for a large population size, it is clear that the corrected estimator tends towards the classical estimate $\hat{V}_{x_i}$.

To illustrate the coupling between RBD, FAST and FEM (rFAST-FE), a post-processing procedure is provided below. Notice that the algorithm 2 gives an implementation of the sensitivity index associated to the random variable $x_i$. The design mapping proposed in [36] can be computed by applying the algorithm 2 simultaneously to each element of the random variable vector.
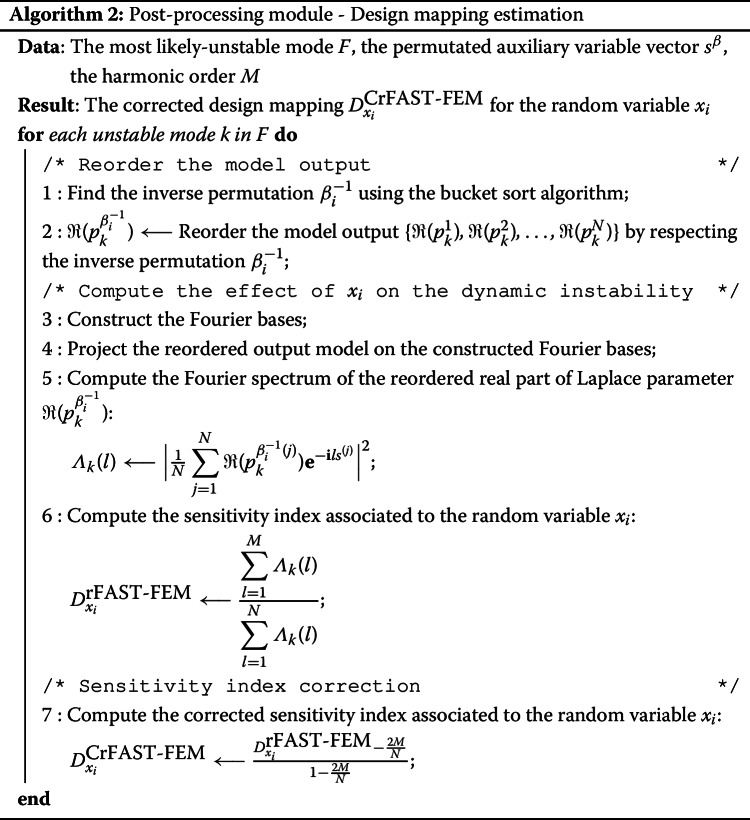


## Numerical analysis of the suggested solver

This section is devoted to illustrate the performances of the rFAST-FE solver through a reduced brake model. First, the uncertainty arising from the tribological aspect involving the friction coefficient is analyzed. Here, authors critically examine the so-called Kolmogorov–Smirnov test procedure combined with the parametric estimation. The results of the latter are compared with the data-driven approach proposed in this paper. Second, the convergence rate of the rFAST-FE solver is confronted to that of the MC-FE and FAST-FE developed in [36]. Finally, an adjustment of the proposed solver, through the order of the harmonics *M* and a less biased sensitivity index estimation, is discussed allowing to find a good trade-off between the computational cost and the prediction accuracy.

### Data-driven random generator validation

It was argued in [18, 31, 50] that the uncertainties play a major role on the prediction of the instabilities. These results led researchers to use random variables to model material properties, friction coefficient and contact interfaces. The methods proposed by Zhang et al. [18] and Chevillot et al. [50] use the so-called “two-sample Kolmogorov-Smirnov” test and the parametric estimation to model the random variables’ PDF. The former quantifies the distance between the EDF of the measured data and the reference CDF, while the latter assumes the shape of the PDF and then, estimates its statistics (e.g. the mean and the variance). Sometimes the above methods are combined. In this case, the “two-sample Kolmogorov-Smirnov” test used to estimate the shape of the probability distribution and the parametric estimation computes its parameters. With no doubt, the aforementioned methods give good approximations of the first two statistical moments, but what about the shape of the distribution which is, in advance, an unknown of the problem? What about the efficiency of the constructed random generator? Will the latter be able to reproduce the statistical behavior of the measured population and thus, integrate in a reliable manner the generated data into the SFE simulations?

To address the above questions, a brake-dyno measured friction coefficient data set is used, with a similar procedure and experimental setup to those used by Zhang et al. [18]. The measured results are separated into cold and hot sections depending on the temperature condition. The goal of this section is to compare Zhang et al. [18] approach with the data-driven scheme coupled with RBD (see “Stochastic pre-processing” section).

**Fig. 1:**
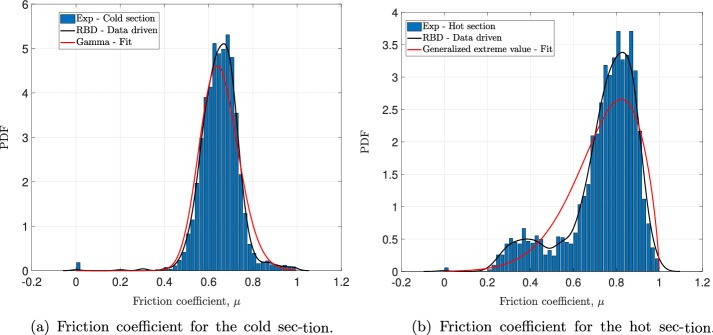
Statistic behavior of the friction coefficient for the cold and hot sections. Comparison between the experiment through the histogram (reference), Kolmogorov–Smirnov test combined with the parametric estimation and the data-driven approach combined with RBD

The statistical behavior of the friction coefficient measured in hot and cold conditions is described through the PDF, as shown in Fig. 1 where the histogram, representation of test data, is compared with the suggested approach (black curves) and the so-called “two-sample Kolmogorov–Smirnov” combined with the parametric estimation (red curves). Indeed, from the experimental data two quantities have been calculated: the EDF and the histogram. The EDF approximates the CDF while the histogram approximates the PDF. The classical approach of modelling uncertainties are successfully applied on the raw measurement data through the computed histogram. The results in Fig. 1 show that the Gamma and the Generalized Extreme Value (GEV) distribution functions represent an acceptable approximation for the friction coefficient of the cold and hot section, respectively. Note that the probability laws mentioned above are adjusted in order to reproduce the histogram. As presented in algorithm 1, the proposed approach considers only the computed EDF. By inverting the latter, while avoiding the singularities that could occur, the RBD-Data-driven random generator was built by explicitly integrating the statistical behavior of the measured data. Once the random generator is constructed, 1000 realizations, for the hot and the cold section, were sampled. Here, the goal is to reconstruct the PDF (black curve) and compare it with the histogram and the analytical PDFs determined by the parametric estimation method. From Fig. 1, one can observe that the fit-based solution using Gamma and GEV PDFs does not agree with the reference (the histogram). For the friction coefficient of the cold section, the histogram is nearly approximated using Gamma PDF, but the latter fails to capture the histogram peak. Another interesting point, observed on the friction coefficient of the hot section, is that its statistic behavior involves clearly a bi-modal distribution which is a non-usual one. Hence, it seems that this non-conventional behavior tends to confirm that the consideration of the so-called usual probabilistic laws are not efficient to model the observed uncertainties since the shape of the histogram is missed using the adjusted GEV PDF. As far as the proposed approach is concerned, it is clear that it performs better than its counterpart. The RBD-DD-based estimations in Fig. 1a and b look qualitatively the same as the computed histograms.

In order to corroborate the results and the above discussion drawn from Fig. 1, 4 statistical indicators will be evaluated, namely the mean, the standard deviation, the skewness and the kurtosis. Hereafter, if not mentioned differently, the 4 indicators will be derived directly from the analytical PDF for the parametric estimation approach, from the reconstructed PDF for our approach (i.e. RBD-DD) and directly from the experimental measurements using the unbiased estimators. They can be written as,

35 $$\begin{aligned} \bar{x}= & {} \frac{1}{N} \displaystyle \sum _{j=1}^{N} x_i^{j}, \end{aligned}$$

36 $$\begin{aligned} s^2= & {} \frac{1}{N-1} \displaystyle \sum _{j=1}^{N} ( x_i^{j} - m )^2, \end{aligned}$$

37 $$\begin{aligned} \mu _3= & {} \frac{\sqrt{N(N-1)}}{N-2} \frac{\frac{1}{N} \displaystyle \sum _{j=1}^{N} (x_i^{j} - m)^3}{\sigma ^3} \end{aligned}$$

and

38 $$\begin{aligned} \kappa = \frac{N-1}{(N-2)(N-3)} \left( (N+1) \frac{\frac{1}{N} \displaystyle \sum _{j=1}^{N} (x_i^{j} - m)^4}{\sigma ^4} - 3(N-1) \right) + 3 \end{aligned}$$

where $\bar{x}$, *s*, $\mu _3$ et $\kappa$ denote the unbiased mean, standard deviation, skewness and kurtosis. $x_i^j$ is the *j*-drawn element of the random variable $x_i$ and *N* refers to the size of the statistic population. Note that the unbiased estimators of the measure defined above are considered as a reference. In what follows, they will be compared to those derived from (i) the parametric estimation and (ii) the RBD-DD approach.

**Fig. 2:**
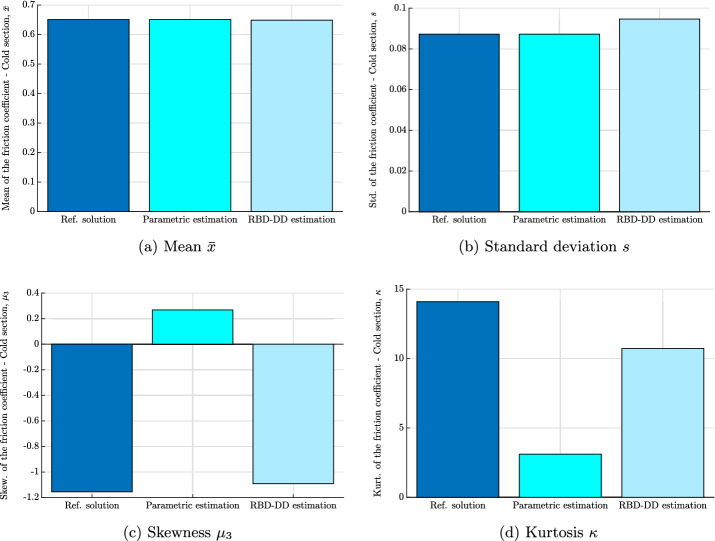
The first four statistic moments for the friction coefficient of the cold section: **a** mean value, **b** standard deviation, **c** skewness and **d** kurtosis. The four statistic indicators are computed directly from (i) the measured data using Eqs. (35), (36), (37) and (38) (the reference solution), (ii) the parametric estimation using the fitted Gamma PDF and (iii) the reconstructed PDF based on the RBD-DD random generator

**Fig. 3:**
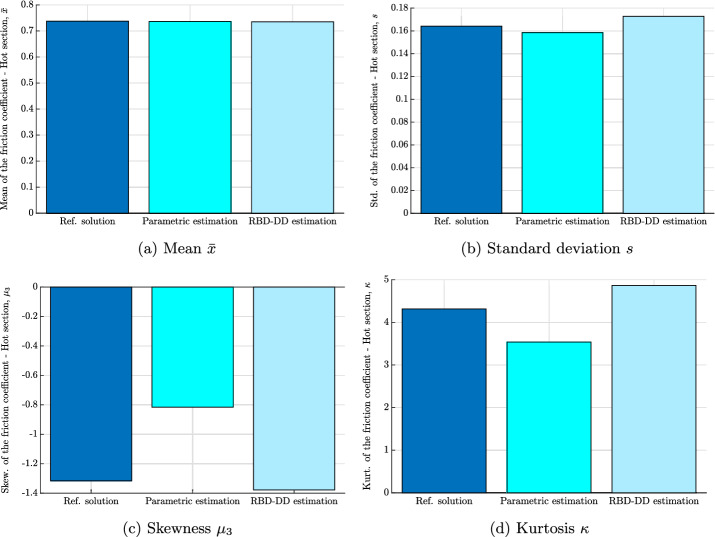
The first four statistic moments for the friction coefficient of the hot section: **a** mean value, **b** standard deviation, **c** skewness and **d** kurtosis. The four statistic indicators are computed directly from (i) the measured data using Eqs. (35), (36), (37) and (38) (the reference solution), (ii) the parametric estimation using the fitted Gamma PDF and (iii) the reconstructed PDF based on the RBD-DD random generator

The finding of the comparative results in Figs. 2 and 3 calls for several remarks and observations. First of all, let us note the similarity in the estimation of the mean and the standard deviation by the RBD-DD approach and that of the parametric estimation (see Figs. 2a, b, 3a and b): this first observation can be explained by the equivalence in terms of performance between the two approaches when estimating the first two statistical moments. Next, let us notice the comparison of the results of the shape factors (i.e. skewness and kurtosis). Indeed, it is clear from Figs. 2c, d, 3c and d that the RBD-DD approach performs better and therefore, reproduces the experimental estimates of skewness and kurtosis for the friction coefficient of the hot and cold section. A difference is noticeable when estimating the kurtosis for the friction coefficient of the hot section (see Fig. 3d) but it remains comparable to the reference and much better than the parametric estimate. Qualitatively, the results of the RBD-DD approach in Figs. 2c and 3c, confirm the asymmetry of the histograms in Fig. 1. Moreover, they indicate that the real probabilistic laws of the experimental measurements are characterized by tails spread to the left, synonymous of a negative skewness coefficient. As for the kurtosis of Figs. 2d and 3d, the RBD-DD-based estimations reveal a *leptokurtic* nature of the friction coefficient of the hot and cold section. These results indicate that the probability laws, corresponding to the measurements, present higher peaks and higher, larger and heavier tails when compared with a Gaussian distribution. With regards to the parametric estimation method, the results of the skewness are not consistent with the measurement and those predicted by the RBD-DD approach. This trend was quite predictable especially for the Gamma fit since its analytic skewness is strictly positive. So, even if the PDF is fitted to the histogram, the skewness will not fit the trend of the measurements.

### Solver performances

**Fig. 4:**
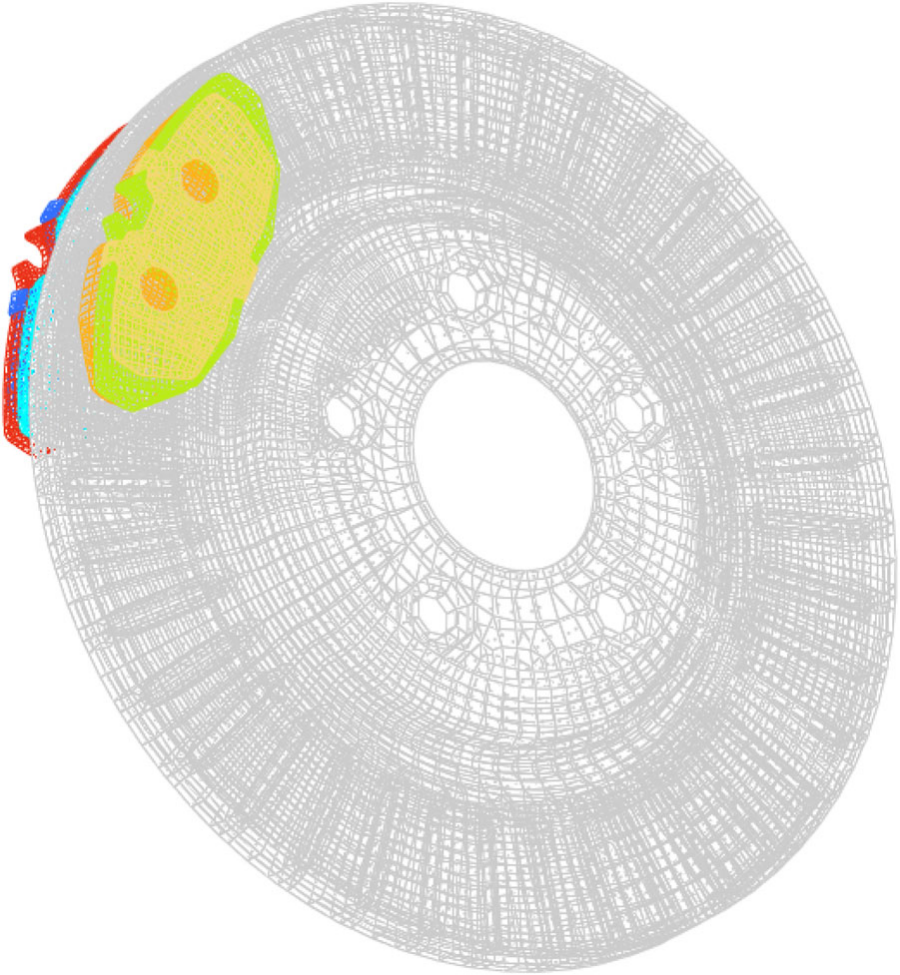
FE model of the reduced brake system

For illustration purposes, the proposed solver named rFAST-FE, described in “Data-driven stochastic complex eigenvalue analysis” section, is applied to a reduced braking system. The latter consists mainly of a rotating part (i.e. the disc) and two pads that come in contact with the disc (see Fig. 4). Each pad is an assembly of 3 other parts: (i) the lining which brakes the disc, (ii) the backplate as a lining support and (iii) the shim which is used as a vibration reducing damper. The frictional contact is modeled by the penalty algorithm using a linear law combined with Coulomb’s model with constant friction coefficient. The discretization of the master and slave surfaces is done through Surface-To-Surface technique. Also, it is necessary to point out that the deterministic part of the solution (Eqs. (14) to (16)) is performed in the small sliding and deformation framework.

The present section aims to analyze the numerical performance of rFAST-FE when solving friction-induced vibrations problem. For that purpose, 10 random variables are chosen, namely

- friction coefficient $\mu$ between the pads and the disc. It should be noted that the friction coefficient is treated numerically identical for both pads.
- Young modulus of the disc $E_d$, the backplate $E_{bp}$ and the shim $E_s$
- stiffness element $G_{ij}$ of the pads which involve an anisotropic behavior,

to perform stochastic simulations on the reduced brake system. Several intensive simulations are carried out on several stochastic populations ranging from 51 to 50, 001 iterations using an In-house High Performance Computing platform equipped with a total of 128 cores. Since we deal with stochastic simulations, a specified 2-level parallelization scheme is used to provide results within a reasonable computing time. For the sake of clarity, let us suppose a SFE model with 3200 iterations and each iteration requires 4 cores. The first level consists on distributing the stochastic iterations on 32 blocks of 4 cores. On the second level, each iteration is parallelized, to solve the deterministic problem, using a shared memory paradigm.

The numerical performances of the rFAST-FE solver will be compared with those of the classic MC-FE and FAST-FE developed in [36, 42]. For a fair comparison, the data-driven framework will not be used here; the same usual laws will be used to model the random variables mentioned above. Additional informations about the stochastic setup are given in [36, 42]. Note also that the order of the harmonics, *M*, is set to 4 for both rFAST-FE and FAST-FE solvers. The investigation of the effect of the parameter *M* on rFAST-FE’s numerical performances will be discussed in “Effect of the harmonic order *M*” section.

**Fig. 5:**
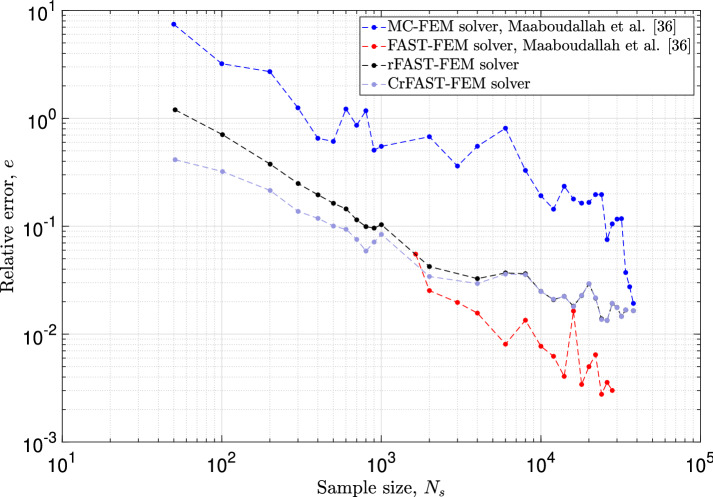
Relative error for the stochastic finite element approaches. Comparison of the relative error in Eq. (39) between (i) MC-FE, (ii) FAST-FE, (iii) rFAST-FE and (iv) the corrected rFAST-FE approach

In order to quantify the convergence rate and compare it with its counterparts (i.e. MC-FE and FAST-FE approaches), the relative error in the Frobenius sense will be defined. It measures the distance of the design mapping introduced in [36] between the finest model and those containing a moderate stochastic population. For example, the finest model for rFAST-FE solver is characterized by a stochastic iteration number of 50001, while the benchmarks for MC-FE and FAST-FE contain 40000 and 30001 iterations, respectively. These represent the reference solution for each method and denoted by $D_{\text{ ref }}$. The relative error reads,

39 $$\begin{aligned} e_i = \frac{\parallel D_{\text{ ref }} - D_{N_i} \parallel _F}{\parallel D_{\text{ ref }} \parallel _F}, ~~~\text{ and }~~ \parallel A \parallel _F = \left( \displaystyle \sum _{i=1}^{k} \sum _{j=1}^{n} \mid a_{ij} \mid ^2 \right) ^{\frac{1}{2}}, ~~ A \in \mathcal {M}_{k\times n}(\mathbb {R}), \end{aligned}$$

and its results are reported in Fig. 5. As mentioned in the literature, the MC-FE approach converges slowly and requires a large number of stochastic iterations to have a meaningful result. On the other hand, it is clear that FAST-FE (red curve) performs better. It was also argued that the convergence of the latter is ensured by the criterion in Eq. (10) which links, in an indirect manner, the CPU time with the dimensionality of the *x*-space $\mathbb {K}^n_x$. In other words, if the number of the random variable increases, the set as well as the maximum value of the characteristic frequencies will increase, leading to a magnification in the stochastic iterations and thus, in the CPU time. Despite all the advantages of FAST-FE mentioned in [36], there remains the black spot of the characteristic frequencies which links the computation time with the dimensionality of the problem. One way to overcome this major drawback is to use the RBD approach coupled with the FAST algorithm. Indeed, the use of unit frequencies combined with random permutations allows to explore what happens below the FAST-FE criterion as illustrated in Fig. 5 (black curve). Using the same stochastic and FE setups, several simulations were conducted by the rFAST-FE approach. Then, the estimator of the error in Eq. (39) is evaluated for each model corresponding to each statistical population. We note that the relative error of rFAST-FE becomes negligible after a sample of 801 iterations, well ahead of that of the MC-FE solver, which requires at least 10000 iterations to reach the same precision. Thus, for the same $10\%$ error prediction, rFAST-FE needs only 801 iterations instead of several thousand. Note, however, that the contribution of the rFAST-FE approach induces an important error when the number of iterations is small. For instance, the prediction with 51 iterations induces an error that exceeds $100\%$ (!). This result agrees with the demonstrated quantification of the estimator presented in Eq. (33). Indeed, when *N* tends to 0, the estimation of the partial variance and thus of the design mapping will be biased. The bias effect was corrected by re-estimating the sensitivity indices over the design mapping using the correction in Eq. (34). At the second step, the above relative error was evaluated on the corrected data (grey curve). As expected, for a large population size, the corrected estimation tends towards the classical biased estimate which is, again, in a good agreement with the theoretical results in Eq. (34). However, when the number of iterations becomes small, the correction introduced above improves the results by reducing the error by half. For example, the relative error at iteration 51 and 101 decreases from $120\%$ and $70\%$ to $41\%$ and $32\%$, respectively.

Finally, let us look at the accuracy of the results predicted by the rFAST-FE solver. To do so, let us take the relative error defined in Eq. (39) but this time the reference will be the solution of the FAST-FE solver on the finest sample containing 30, 001 iterations. At the i-th stochastic model the above error is calculated, in Frobenius sense, as:

40 $$\begin{aligned} \varepsilon _i = \frac{\parallel D^{\text{ FAST-FE }}_{\text{ ref }} - D^{\text{ rFAST-FE }}_{N_i} \parallel _F}{\parallel D^{\text{ FAST-FE }}_{\text{ ref }} \parallel _F}. \end{aligned}$$

**Fig. 6:**
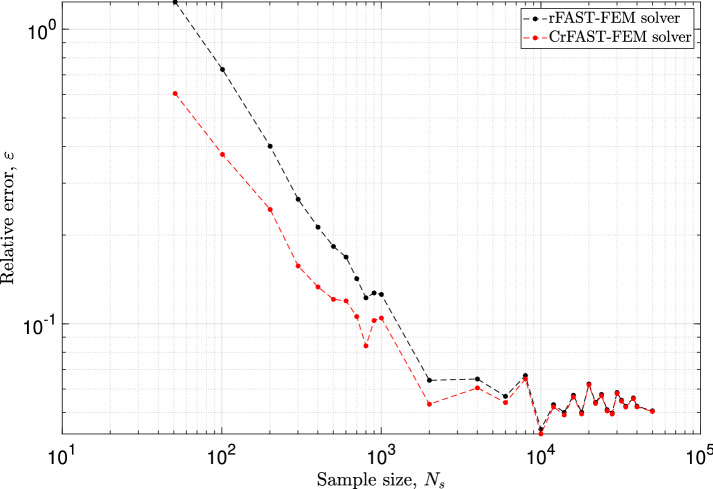
Evolution of the relative error of Eq. (40) for the classical (black curve) and corrected estimation (red curve) of the rFAST-FE solver

**Fig. 7:**
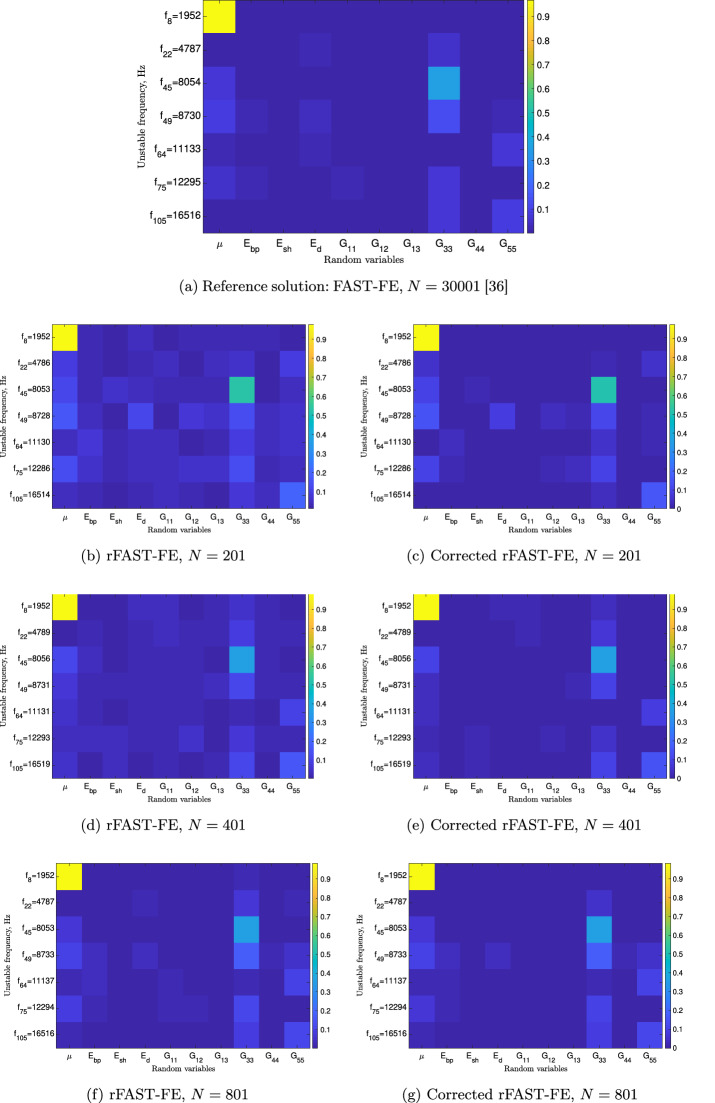
Design mapping results—effect of the sample size *N* and the weighted correction on the response design map: Top, **a** represents the reference solution predicted by FAST-FE approach using 30, 001 iterations. The second, third and fourth lines show a comparison of rFAST-FE prediction with the corrected one for a sample size of 201 (**b**, **c**), 401 (**d**, **e**) and 801 (**f**, **g**), respectively

Fig. 6 presents the evolution of the error quantifying the distance between the reference solution, given by FAST-FE, and each stochastic rFAST-FE model with and without the proposed correction process. The obtained results confirm again the previous observations. Thus, the weighted correction introduced in Eq. (34) allows to obtain acceptable results with a coarse model. In addition to the analysis conducted earlier on the accuracy of rFAST-FE compared to its counterpart (i.e., FAST-FE), it is also interesting to observe closely the design mappings obtained with a reduced iteration number. Figure 7 shows comparative results between the reference solution (Fig. 7a) obtained by the FAST-FE approach using 30001 iterations and those obtained by rFAST-FE with and without correction process. The comparison of the resulted design mappings, without correction (Figs. 7b, d, f), with the reference shows that the accuracy at the prediction level increases with the number of iterations, going from 201 up to 801. On the other hand, the weighted correction significantly improves the results. With only 201 iterations, we find approximately the same map as the reference. Indeed, the corrected results of Fig. 7c shows that there are 7 instabilities (i.e., 7 unstable modes) which result from the contribution of the friction coefficient, $\mu$, and the stiffness $G_{33}$ of pads exactly as discussed in [36, 42]. Using the rFAST-FE solver with the correction procedure reduces significantly the computational cost. Indeed, the results in Fig. 7c require a CPU cost that does not exceed 1h on 128 cores, which is 7 times lower than that of FAST-FE which converges after 1641 iterations (see the criterion of Eq. (10)).

### Effect of the harmonic order *M*

Since Fourier expansion is introduced within FEM, the choice of the harmonic order *M*, used to compute the partial variances, will affect the design mapping results. In the previous “Solver performances” section, the harmonic order is kept the same, namely $M=4$, for both FAST-FE and rFAST-FE solvers. The previous configuration comes from the literature where authors have argued, without a strong demonstration, that an order M of 4 or 6 is generally considered sufficient [51, 52]. In the most recent works [53–55], authors do not even specify (!) the order of harmonics used to estimate the partial variances, while others prefer to make it at the disposition of the investigator [44].

This section shows how the order of harmonics *M* affects the predicted design mapping results for rFAST-FE solver. First of all, a set of the harmonic order is considered by going from 1 to 10. Then, for each value, errors in Eqs. (39) and (40) are computed following the correction procedure outlined previously.

**Fig. 8:**
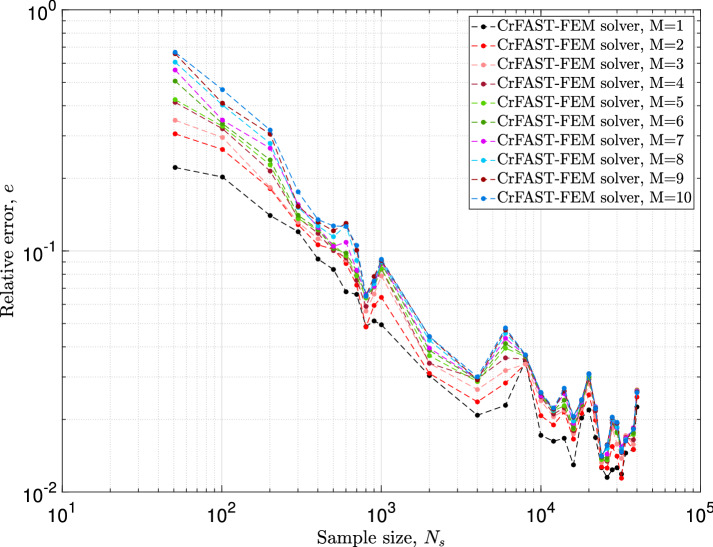
The effect of the order of the harmonies, *M*, on the error of Eq. (39) taking into account the correction process introduced by Eq. (34)

**Fig. 9:**
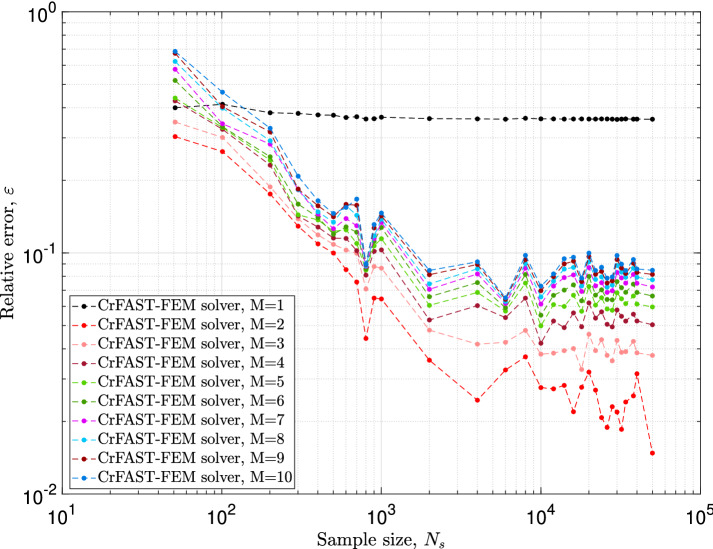
The effect of the order of the harmonies, *M*, on the error of Eq. (40) taking into account the correction process introduced by Eq. (34)

In the first of these, the computed relative error in Eq. (39), see Fig. 8, reveals an improvement of the convergence behavior when the harmonies order decreases. Indeed, under the correction procedure, a harmonic order of 1 lead to significant error reduction for a small sample size models. In the second, i.e. the relative error of Eq. (40) in Fig. 9, one can observe a non-trivial behavior. First of all, when the harmonies order is equal to 1, the computed error remains higher, $40\%$, for each stochastic model, synonymous of the existence of a large difference between the reference solution (i.e. FAST-FE on 30, 001 iterations with $M=4$) and the corrected results predicted by the suggested approach. It should be noted, however, that this no-conventional behavior vanishes with the increase of the so-called harmonic order. Overall, the error decreases from $68\%$ at 51 iterations to $8\%$ at 2001 with an evident asymptotic behavior starting from 2001 samples. It is also important to note and observe the error evolution with a harmonic order of 2 for both Figs. 8 and 9. According to the red curve of the above figures, it is immediately evident that the corrected solver rFAST-FE using an harmonic order of 2 is super-convergent and hence, gives much inferior errors for all the stochastic models. For instance, we notice immediately that the estimated error decreases from 30 to $3\%$ from 51 iterations to 2001. This performance comparison, under the above conditions *i.e. correction procedure using an harmonies order of 2*, is quite remarkable and efficient for this type of problem. It allows to predict instabilities through the so-called design mapping within very reasonable stochastic iterations. Furthermore, the last point is completely dissociated from the dimensionality of the problem, which will avoid the “curse of dimensionality” occurring when using high-dimensional spaces.

## Conclusion

Throughout this paper, we have addressed multiple aspects pertaining to the prediction of instabilities of a frictional system. First, using a data-driven framework smoothed by the Random-Balance-Design approach, the observed uncertainties can be modeled efficiently whether the probability law is conventional or not. The DD-RBD procedure has proved to be a very powerful stochastic preprocessor tool leading to recover, through the estimated CDF, the experimental stochastic behavior of the random variables. Second, the introduction of rFAST algorithm combined with FEM allows to drop out the characteristic frequencies and hence, explore what happens out of the box without, necessarily, respecting the tricky FAST-FE criterion which involves indirectly the dimensionality of the problem. Using the same stochastic and FE setups as Maaboudallah et al. [36], investigations have shown that the novel approach performs better than MC-FE. However, compared with the classic FAST-FE, the latter reduces errors when the stochastic iterations are beyond FAST-FE criterion. On the other hand, when the latter limits the leeway, rFAST-FE makes it possible to lighten the constraints and thus, to perform stochastic finite element simulations with a lower iteration number.

On the other hand, we have demonstrated that the partial variance estimation is biased. It has been found that the bias term contains the sample size, *N*, as well as the residual partial variance $V_{\sim x_i}$. It is important to note that these theoretical results agree very well with the computed error, i.e. when the stochastic iterations decrease, the bias as well as the relative error increase. In order to overcome the bias effect, a weighted correction is introduced on the estimated partial variance. In this manner, the errors committed at lower stochastic iterations are reduced by a half.

In the last part of the paper, authors examine the effect of the harmonic order on the computed errors. A harmonic order of 2 was found the best candidate to efficiently reduce the errors of the corrected rFAST-FE solver.

We expect that these coupled approach which involves (i) DD-RBD preprocessing tool, (ii) rFAST-FE solver and (iii) the correction process will enhance engineers’ capabilities to solve friction-induced vibration. The contribution of this study is flexible and can be adapted with any off-the-shelf deterministic solver whether open-source or not.

## Appendix A: Decomposition of the Fourier spectrum estimator based on the RBD-FAST approach

For the purpose of quantifying the error in the estimation of partial variances related to the main effect, let us rewrite the model in the following way,

41 $$\begin{aligned} \Re (p_k) = \text{ FEM } (x_i) + \varrho _{\sim x_i}. \end{aligned}$$

Note that the term $\varrho _{\sim x_i}$ involves the contribution of the random variable vector *x* except $x_i$, while $\text{ FEM }(x_i)$ is resulted from the main effect. It is important to note, that the last expression comes from the so-called high dimensional model representation (HDMR), without taking into account the sum, under Sobol’s assumptions [56]. We write,

42 $$\begin{aligned} \Re (p_k) = h_0 + \displaystyle \sum _{i} h_i(x_i) + \displaystyle \sum _{i<j} h_{ij}(x_i,x_j) + \dots + h_{12 \dots m} (x_1, x_n, \dots , x_n), \end{aligned}$$

where the decomposed terms are given by:

43 $$\begin{aligned} \left\{ \begin{array}{c} h_0 = E(\Re (p_k)),\\ h_i = E(\Re (p_k) \mid x_i) - E(\Re (p_k)), \\ h_{ij} = E(\Re (p_k) \mid x_i,x_j) - f_i - f_j - E(\Re (p_k)), \\ \text{ and } \text{ so } \text{ on. } \\ \end{array} \right. \end{aligned}$$

Since the random generator is periodic, random variables will be sampled periodically. For that logic, the following developments will be presented directly on the $\theta$-space using the composite function $\text{ FEM } \circ G$.

First, let us examine the mean of the second term in Eq. (41), namely $\varrho _{\sim x_i}$. The linearity in the expected value allows us to write

44 $$\begin{aligned} E(\varrho _{\sim \theta _i}) = E(\Re (p_k)) - E(\text{ FEM } \circ G (\theta _i)). \end{aligned}$$

To lighten the mathematical development, $\text{ FEM } \circ G (\theta _1, \dots , \theta _n)$ will be replaced by $\Re (p_k)$ in what follows. Therefore, the expected value of $\text{ FEM } \circ G (\theta _i)$ is given by,

45 $$\begin{aligned} \begin{aligned} E(\text{ FEM } \circ G (\theta _i))&= E \left( E\left( \text{ FEM } \circ G (\theta _1, \dots , \theta _n) \mid \theta _i \right) \right) \\&= E \left( E\left( \Re (p_k)\mid \theta _i \right) \right) \\&= \int _{\mathbb {R}} E\left( \Re (p_k) \mid \theta _i \right) f_{\theta _i} \, d \theta _i \\&= \int _{\mathbb {R}} \int _{\mathbb {R}} \Re (p_k) f_{\Re (p_k) \mid \theta _i} \, d \Re (p_k) f_{\theta _i} \, d \theta _i \\&= \int _{\mathbb {R}} \Re (p_k) \int _{\mathbb {R}} f_{\Re (p_k)} \, d \Re (p_k) \, d \theta _i \\&= E(\Re (p_k)), \end{aligned} \end{aligned}$$

where $f_{\Re (p_k) \mid \theta _i}$ is the conditional PDF for the random variable $\Re (p_k)$ given $\theta _i$. Hence, according to Eq. (44), the expected value of the residual part $\varrho _{\sim \theta _i}$ is,

46 $$\begin{aligned} E(\varrho _{\sim \theta _i}) = 0. \end{aligned}$$

Second, lets us consider the estimation in Eq. (20), but the concerns will be dedicated only to the main effect of the random variable $x_i$ (equivalently, $\theta _i$). Mathematically, we write,

47 $$\begin{aligned} C^{(\theta _i)}_{0, \dots ,0, \gamma _i, 0, \dots ,0} = \frac{1}{2 \pi } \int _{-\pi }^{\pi } \text{ FEM } \circ G (\theta _1, \dots , \theta _n) e^{-\textbf{j}(\gamma _i \theta _i)} \, d \theta _i, \end{aligned}$$

which can be rewritten by

48 $$\begin{aligned} C^{(\theta _i)}_{0, \dots ,0, \gamma _i, 0, \dots ,0} = E_{\theta _i} \left( \text{ FEM } \circ G(\theta _i) e^{-\textbf{j}(\gamma _i \theta _i)} \right) , \end{aligned}$$

where,

49 $$\begin{aligned} \text{ FEM } \circ G(\theta _i) = E\left( \text{ FEM } \circ G (\theta _1, \dots , \theta _n) \mid \theta _i \right) . \end{aligned}$$

Numerically, Eq. (47) can be computed on the model output using the rectangle rule over *N* samples. Thus, its estimate is given by,

50 $$\begin{aligned} \hat{C}^{(\theta _i)}_{0, \dots ,0, \gamma _i, 0, \dots ,0} = \frac{1}{N} \displaystyle \sum _{j=1}^{N} \text{ FEM } \circ G (\theta _1^{(j)}, \dots , \theta _n^{(j)}) e^{-\textbf{j} \gamma _i \theta _i^{(j)}}. \end{aligned}$$

It is important to note that the *j*th-element $\text{ FEM } \circ G (\theta _1^{(j)}, \dots , \theta _n^{(j)})$ in the estimation of Eq. (50) contains the main effect and also the residual $\varrho _{\sim \theta _i}$. Following that, the previous estimation can be decomposed into two part,

51 $$\begin{aligned} \hat{C}^{(\theta _i)}_{0, \dots ,0, \gamma _i, 0, \dots ,0} = \hat{C}^{(\theta _i), FEM\circ G}_{0, \dots ,0, \gamma _i, 0, \dots ,0} + \hat{C}^{(\theta _i), \varrho _{\sim \theta _i}}_{0, \dots ,0, \gamma _i, 0, \dots ,0}, \end{aligned}$$

where

52 $$\begin{aligned} \hat{C}^{(\theta _i), FEM\circ G}_{0, \dots ,0, \gamma _i, 0, \dots ,0} = \frac{1}{N} \displaystyle \sum _{j=1}^{N} \text{ FEM } \circ G (\theta _i^{(j)}) e^{-\textbf{j}\gamma _i \theta _i^{(j)}} \end{aligned}$$

and

53 $$\begin{aligned} \hat{C}^{(\theta _i), \varrho _{\sim \theta _i}}_{0, \dots ,0, \gamma _i, 0, \dots ,0} = \frac{1}{N} \displaystyle \sum _{j=1}^{N} \varrho _{\sim \theta _i}^{(j)} e^{-\textbf{j}\gamma _i \theta _i^{(j)}}. \end{aligned}$$

As explained in “Solver performances” section, the error committed in estimation Eq. (52) is of the order of $\frac{1}{N^2}$. However, the error of estimate Eq. (53) remains unknown. To evaluate it, the Central Limit theorem will be applied on Eq. (53). Thus, by evaluating the complex random variable $\varrho _{\sim \theta _i} e^{-\textbf{j}\gamma _i \theta _i}$, the Fourier coefficient in Eq. (53) will nearly follow a Gaussian law. We have

54 $$\begin{aligned} \hat{C}^{(\theta _i), \varrho _{\sim \theta _i}}_{0, \dots ,0, \gamma _i, 0, \dots ,0} \sim \mathcal {N}(m,\frac{\sigma ^2}{N}), \end{aligned}$$

where $m = 0$ is the mean value and $\sigma ^2 = V(\varrho _{\sim \theta _i})$ refers to the variance of the residual part.

Hence, the error of the estimation in Eq. (53) decays at a rate of $\frac{1}{\sqrt{N}}$ relatively higher than that of the estimation in Eq. (52).

## Appendix B: Evaluation of the quality of the partial variance estimator based on the RBD approach

Let consider the decomposition in Eq. (51). By using the fact that the estimation $\hat{C}^{(\theta _i), FEM\circ G}_{0, \dots ,0, \gamma _i, 0, \dots ,0}$ has a negligible error. One can approximate $C^{(\theta _i)}_{0, \dots ,0, \gamma _i, 0, \dots ,0}$ directly by its estimate $\hat{C}^{(\theta _i), FEM\circ G}_{0, \dots ,0, \gamma _i, 0, \dots ,0}$. Therefore, Eq. (51) can be rewritten in terms of the mean as follows,

55 $$\begin{aligned} E\left[ \mid \hat{C}^{(\theta _i)}_{0, \dots ,0, \gamma _i, 0, \dots ,0} \mid ^2\right] = E\left[ \mid C^{(\theta _i)}_{0, \dots ,0, \gamma _i, 0, \dots ,0} \mid ^2\right] + E\left[ \mid \hat{C}^{(\theta _i), \varrho _{\sim \theta _i}}_{0, \dots ,0, \gamma _i, 0, \dots ,0} \mid ^2 \right] . \end{aligned}$$

On the one hand, the estimation in the second term of Eq. (55) can be decomposed into the cosine and sinus basis. We write,

56 $$\begin{aligned} \hat{C}^{(\theta _i), \varrho _{\sim \theta _i}}_{0, \dots ,0, \gamma _i, 0, \dots ,0} = \hat{a}^{(\theta _i), \varrho _{\sim \theta _i}}_{0, \dots ,0, \gamma _i, 0, \dots ,0} - \textbf{i} \hat{b}^{(\theta _i), \varrho _{\sim \theta _i}}_{0, \dots ,0, \gamma _i, 0, \dots ,0}, \end{aligned}$$

where

57 $$\begin{aligned} \hat{a}^{(\theta _i), \varrho _{\sim \theta _i}}_{0, \dots ,0, \gamma _i, 0, \dots ,0} = \frac{1}{N} \displaystyle \sum _{j=1}^{N} \varrho _{\sim \theta _i}^{(j)} \cos (\gamma _i \theta _i^{(j)}) \end{aligned}$$

and

58 $$\begin{aligned} \hat{b}^{(\theta _i), \varrho _{\sim \theta _i}}_{0, \dots ,0, \gamma _i, 0, \dots ,0} = \frac{1}{N} \displaystyle \sum _{j=1}^{N} \varrho _{\sim \theta _i}^{(j)} \sin (\gamma _i \theta _i^{(j)}). \end{aligned}$$

According the CLT, $\hat{a}^{(\theta _i), \varrho _{\sim \theta _i}}_{0, \dots ,0, \gamma _i, 0, \dots ,0}$ and $\hat{b}^{(\theta _i), \varrho _{\sim \theta _i}}_{0, \dots ,0, \gamma _i, 0, \dots ,0}$ follow a Gaussian distribution with a zero mean and a variance of $\frac{V_{\varrho _{\sim \theta _i}}}{2N}$.

Using the fact that

$$\mid \hat{C}^{(\theta _i), \varrho _{\sim \theta _i}}_{0, \dots ,0, \gamma _i, 0, \dots ,0} \mid ^2 = \mid \hat{a}^{(\theta _i), \varrho _{\sim \theta _i}}_{0, \dots ,0, \gamma _i, 0, \dots ,0}\mid ^2 + \mid \hat{b}^{(\theta _i), \varrho _{\sim \theta _i}}_{0, \dots ,0, \gamma _i, 0, \dots ,0} \mid ^2$$

and

$$\begin{aligned} E\left[ \mid \hat{a}^{(\theta _i), \varrho _{\sim \theta _i}}_{0, \dots ,0, \gamma _i, 0, \dots ,0} \mid ^2 \right] = E\left[ \mid \hat{b}^{(\theta _i), \varrho _{\sim \theta _i}}_{0, \dots ,0, \gamma _i, 0, \dots ,0} \mid ^2 \right] = \frac{V_{\varrho _{\sim \theta _i}}}{2N}, \end{aligned}$$

Equation (55) can be rewritten as follows,

59 $$\begin{aligned} E\left[ \mid \hat{C}^{(\theta _i)}_{0, \dots ,0, \gamma _i, 0, \dots ,0} \mid ^2\right] \approx \mid C^{(\theta _i)}_{0, \dots ,0, \gamma _i, 0, \dots ,0} \mid ^2 + \frac{V_{\varrho _{\sim \theta _i}}}{N}. \end{aligned}$$

The last equation shows that the estimation of the Fourier spectrum is biased. The bias depends essentially on the variance of the residual, $\varrho _{\sim \theta _i}$, and the number of stochastic iterations *N*.
